# Lumbar lordosis morphology correlates to pelvic incidence and erector spinae muscularity

**DOI:** 10.1038/s41598-020-80852-7

**Published:** 2021-01-12

**Authors:** Yang Li, Jianmin Sun, Guodong Wang

**Affiliations:** Shandong Provincial Hospital Affiliated to Shandong First Medical University, 9677 Jingshi Road, Jinan, Shandong Province China

**Keywords:** Musculoskeletal system, Spinal cord diseases, Radiography

## Abstract

The retrospective study aimed to investigate the relationship between lumbar lordosis morphology, pelvic incidence and paraspinal muscle. It enrolled asymptomatic adult volunteers aged between 18 and 45 years old. Lumbar lordosis morphology, consisting of total lumbar lordosis (LL), proximal lumbar lordosis (PLL), distal lumbar lordosis (DLL), lumbar lordosis apex (LLA) and inflexion point, was evaluated, as well as pelvic incidence (PI) and muscularity of erector spinae (ES) and multifidus. Pearson correlation was performed to analyze the relationship between each other parameter. Cases were stratified according to pelvic incidence (very low < 30°, low 30°–45°, moderate 45°–60°, and high > 60°), comparison between groups was performed by univariance analysis. 87 asymptomatic adult volunteers (33 females and 54 males) were included in the study. PLL revealed a correlation with LLA (r = 0.603, *p* = 0.002) and inflexion point (r = 0.536, *p* = 0.004), but did not DLL with LL apex (r = 0.204, *p* = 0.058) or inflexion point (r = 0.210, *p* = 0.051). PI revealed a greater correlation with PLL (r = −0.673, *p* < 0.001) than with DLL (r = −0.237, *p* = 0.045). Linear stepwise regression analysis also exhibited the correlation between PI and PLL (R^2^ = 0.452, PLL = 16.2–0.61 * PI, *p* < 0.001). ES muscularity correlated with LL apex (r = −0.279, *p* = 0.014) and inflexion point (r = −0.227, *p* = 0.047). Stratification by PI demonstrated PLL increased across groups (*p* < 0.001), but DLL was comparable between low and moderate PI group (*p* = 0.329). Lumbar lordosis morphology appears to accommodate to pelvic incidence and erector spinae muscularity. Proximal lumbar lordosis has a bigger correlation with pelvic incidence than the distal lumbar lordosis. The results are helpful for restoring a rational lumbar lordosis shape in long fusion surgery.

## Introduction

Sagittal spinal alignment and lumbar lordosis shape varies across individuals^1–5^, especially in different diseases^6–8^. It has been a research hot spot nowadays, since Roussouly classification of normal spinal sagittal profile^9–11^. Roussouly et al. established the classification consisting of 5 types according to pelvic incidence (PI) value^11^: type 1 and 2 with low grade PI and SS(PI < 45°, SS < 35°), type 3 + with low grade PI (PI < 45°) and anteverted pelvis (SS > 35°), type 3 with high grade PI (PI > 45°) and anteverted pelvis (35° < SS < 45°), type 4 with high grade PI (PI > 45°) and over anteverted pelvis (SS > 45°) . Lumbar lordosis (LL) morphology differs among the 5 types, including LL apex, inflexion point, LL value, and lordosis distribution index (LDI), which means the proportion of distal lumbar lordosis (DLL) in global lordosis^11^.

Little attention is paid on proximal lumbar lordosis (PLL), until Pesenti and colleagues illustrate the amount of PLL is related to PI^12^, but DLL remains relatively constant. However, the mechanism how LL morphology accommodates to PI has not been deeply investigated, neither the role PLL plays in it.

Paraspinal muscle is supposed a valuable factor in compensatory mechanism of LL morphology, considering it puts an influence on LL formation in adolescents^13^, as well as on LL degeneration in elderly population^14–16^. We retrospectively studied the sagittal profile and paraspinal muscle condition of 87 asymptomatic young adults, in order to investigate the impact of paraspinal muscle and pelvic incidence on LL morphology and the role of PLL in adjusting LL to them.

## Materials and methods

This was a retrospective comparative study. All methods were performed in accordance with the relevant guidelines and regulations. Written informed consent was obtained from all subjects participated in the study, and ethical permission to conduct this retrospective study was obtained from the Shandong Provincial Hospital ethics committee.

Inclusion criteria: adult healthy volunteers with age between 18 and 45 years, without low back pain or radiculopathy symptoms. Exclusion criteria: 1, with spinal deformity; 2, with lumbar or thoracic disease; 3, with hip joint or pelvic disease; 4, with a history of spinal surgery.

**Figure 1:**
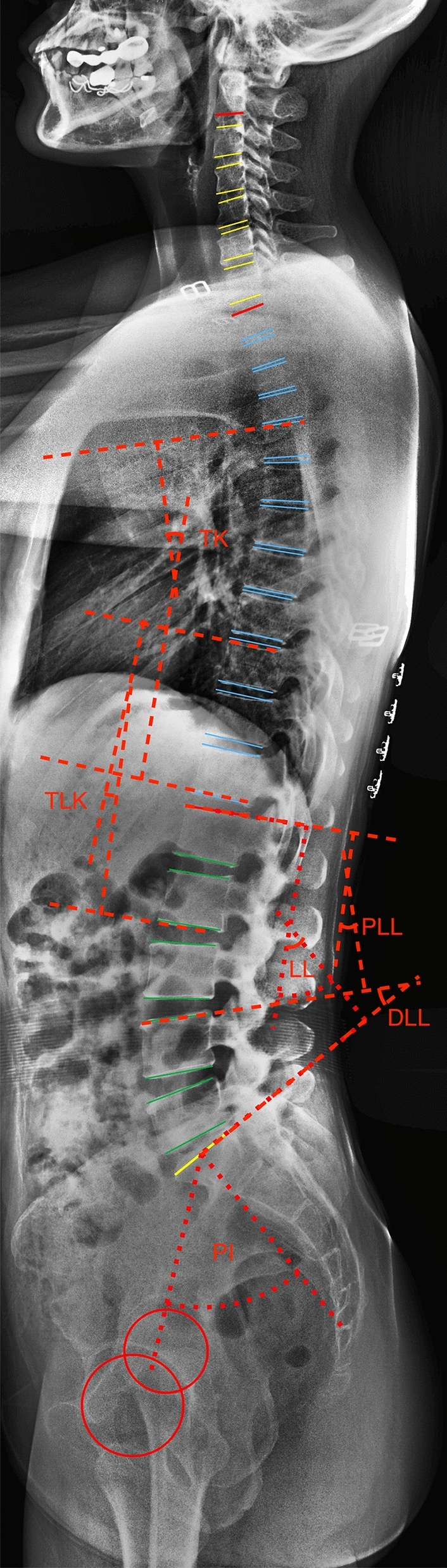
The graph shows the measurement of the sagittal spinopelvic alignments. *PI* pelvic incidence, *LL* lumbar lordosis (L1–S1), *PLL* proximal lumbar lordosis (L1–L4 superior endplate), *DLL* distal lumbar lordosis (L4 superior endplate to S1), *TLK* thoracolumbar kyphosis (T10–L2), *TK* thoracic kyphosis (T5–T12).

Lumbar and pelvic morphology were assessed on lateral 36-in. standing radiographs according to the established positioning protocol (Fig. 1). Pelvic morphology consisted of pelvic incidence (PI), pelvic tilt (PT), and sacral slope (SS). Lumbar morphology included lumbar lordosis (LL), proximal lumbar lordosis (PLL = Cobb angle between L1 and L4 upper endplate), distal lumbar lordosis (DLL = Cobb angle between L4 and S1 upper endplate), lumbar lordosis apex (LLA), and inflexion point. The measurement was processed on Surgimap software (Nemaris Inc., NY, US). Lordosis was recorded as negative and kyphosis was recorded as positive.

**Figure 2:**
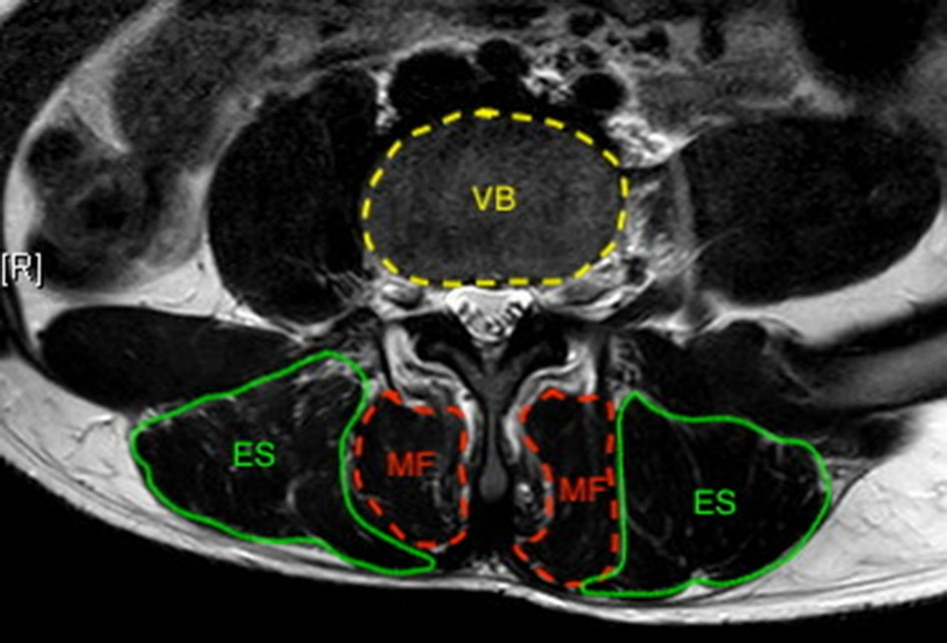
Measurement of cross-sectional area (CSA) of erector spinae (ES) and multifidus (MF) and L4 vertebral body using magnetic resonance imaging. CSA of muscle was calculated as the mean value of bilateral CSA. Muscularity = CSA of muscle − vertebral body ratio.

Erector spinae and multifidus muscularity was defined as the ratio between cross sectional area (CSA) of erector spinae (ES) and multifidus (MF) and CSA of vertebrae body (VB). The CSA of ES and MF was measured on T2-weighted image by outlining the fascial boundary of the muscle using ImageJ software (National Institutes of Health, Maryland, US). CSA of muscle was calculated as the mean value of bilateral CSA. The measurement of ES, MF and VB was performed at L4 inferior endplate level (Fig. 2), as body weight correlated significantly with L4 CSA^17^.

Stratification by PI value was performed: very low < 30°, low 30°–45°, moderate 45°–60°, and high > 60°, according to the normal distribution of PI in the entire cohort which was tested by Kolmogorov–Smirnov test. Independent t test and one-way ANOVA were performed for comparison of quantitative variables between groups, and Chi square test was performed for qualitative variables. Pearson correlation was performed to analyze the relationship between each other parameter. Linear stepwise regression analysis was also conducted for the relationship between quantitative variables. The distribution of parameters was record as mean and standard deviation. The significance threshold was set at 5% (*p* < 0.05). The statistic calculating was processed on SPSS software (IBM Inc., Chicago, IL, US).

### Ethical approval

Permission to conduct this retrospective study was obtained from the authors’ hospital ethics committee.

### Consent to participate

All the patients included consent to participate in the retrospective study.

### Consent for publication

All the patients included, as well as the hospital ethics committee, were consent for publication of the retrospective study.

## Results

A cohort of 100 asymptomatic adult volunteers were recruited between September 2017 and October 2019. Out of them, 6 volunteers were excluded due to spinal deformity or spondylolisthesis, and 7 were excluded due to lumbopelvic transitional vertebrae. Out of 100 volunteers, 87 asymptomatic adult volunteers were included in the study. There were 33 females and 54 males. The mean age was 37.3 ± 7.2 years (range 19–45 years). The mean value of LL was − 39.9° ± 14.5° (range − 5° to − 75°), PLL was − 10.9° ± 10.0° (range 16° to − 38°), DLL was − 29.0° ± 7.9° (range − 7° to − 50°), TLK was 3.6° ± 11.8° (range − 11° to 20°), TK was 25.8° ± 11.8° (range 6°–54°). MF muscularity was 0.34 ± 0.13 (range 0.15–0.69), ES muscularity was 0.98 ± 0.27 (range, 0.49–1.70). According to Roussouly classification, there were 19 cases in type 1 (22%, 19/87), 28 cases in type 2 (32%, 28/87), 29 cases in type 3(33%, 29/87), and 11 cases in type 4(13%, 11/87).

**Figure 3:**
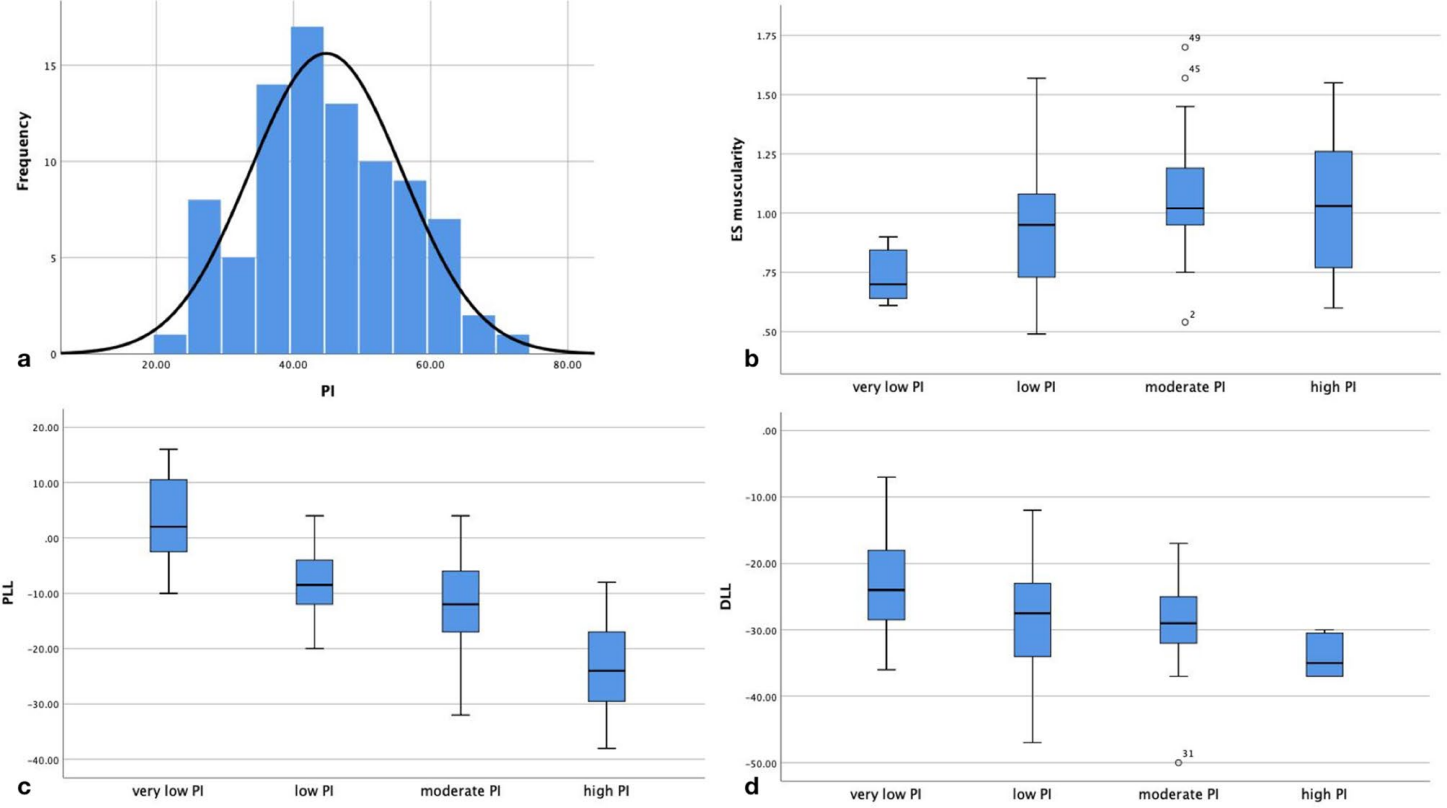
The graphs show the distribution of pelvic incidence (PI) value (**a**), erector spinae (ES) muscularity in PI stratified groups (**b**), proximal lumbar lordosis (PLL) in PI stratified groups (**c**), and distal lumbar lordosis (DLL) in PI stratified groups (**d**).

PI value followed a natural distribution with mean value of 44.8° ± 11.1° (range 22°–73°, 95% CI 42–47) (Fig. 3). The normality was tested by Kolmogorov–Smirnov test (*p* = 0.200). According to the stratification by PI value, there were 9 cases with very low PI (PI < 30°), 36 cases with low PI (PI = 30°–45°), 32 cases with moderate PI (PI = 45°–60°), and 10 cases with high PI (PI > 60°).

Lumbar lordosis morphology differed among the groups (Table 1). Value of LL and PLL increased across groups from very low PI group to high PI group (*p* < 0.001). Very low PI group had a smaller DLL (DLL = −22.9° ± 9.5°) and high PI group had a higher DLL (DLL = −33.8° ± 3.4°), but, as the majority groups, low PI group and moderate PI group had similar DLL (DLL = −27.8° ± 8.0° and − 28.5° ± 6.4°, *p* = 0.329) (Fig. 3). Location of LL apex and inflexion point also inclined across groups from very low PI group to high PI group (*p* < 0.001). No significant difference of MF muscularity existed among the groups, but ES muscularity increased from 0.74 ± 0.12 in very low PI group to 1.07 ± 0.24 in moderate PI group (*p* = 0.019) (Fig. 3). High PI group had a slight lower ES muscularity than moderate PI group, but without statistically significant (*p* = 0.746).

**Table 1:** Univariance analysis of lumbar lordosis morphology parameters.

Parameters	Very low PI	Low PI	Moderate PI	High PI	*p* value
	(< 30°)	(30°–45°)	(45°–60)	(> 60°)	
Case no	9	36	32	10	–
PI (°)	26.9 ± 1.5	38.5 ± 3.5	50.2 ± 4.0	64.1 ± 3.0	< 0.001
PT (°)	11.9 ± 6.5	12.0 ± 5.7	17.7 ± 6.0	21.0 ± 8.6	< 0.001
SS (°)	15.0 ± 7.2	26.5 ± 5.5	32.6 ± 7.2	43.1 ± 8.8	< 0.001
LL (°)	− 19.4 ± 8.1	− 35.7 ± 10.5	− 41.2 ± 11.1	− 57.1 ± 12.6	< 0.001
PLL (°)	3.4 ± 9.3	− 8.0 ± 5.4	− 12.7 ± 7.9	− 23.3 ± 10.9	< 0.001
DLL (°)	− 22.9 ± 9.5	− 27.8 ± 8.0*	− 28.5 ± 6.4*	− 33.8 ± 3.4	0.015
TLK (°)	8.4 ± 11.0	4.6 ± 5.2	0.7 ± 6.1	3.7 ± 7.4	0.031
TK (°)	12.4 ± 8.3	25.1 ± 11.4*	24.1 ± 9.0*	30.7 ± 13.6	0.004
ES muscularity	0.74 ± 0.12	0.95 ± 0.26	1.07 ± 0.24	1.03 ± 0.35	0.019
MF muscularity	0.30 ± 0.10	0.36 ± 0.15*	0.33 ± 0.9*	0.34 ± 0.14	0.663
**LL Apex**					
L3	0	0	6	6	< 0.001
L3/4	0	1	9	4	
L4	0	18	15	0	
L4/5	0	12	2	0	
L5	9	5	0	0	
**Inflexion point**					
T10	0	0	2	0	< 0.001
T11	0	0	3	3	
T12	0	3	10	4	
L1	0	21	15	3	
L2	3	10	2	0	
L3	6	2	0	0	

*NO.* number, *PI* pelvic incidence, *PLL* proximal lumbar lordosis, *DLL* distal lumbar lordosis, *LL Apex* lumbar lordosis apex, *ES* erector spinae, *MF* multifidus.

*Without significance (*p* > 0.05) when compared between low PI group and moderate PI group.

**Figure 4:**
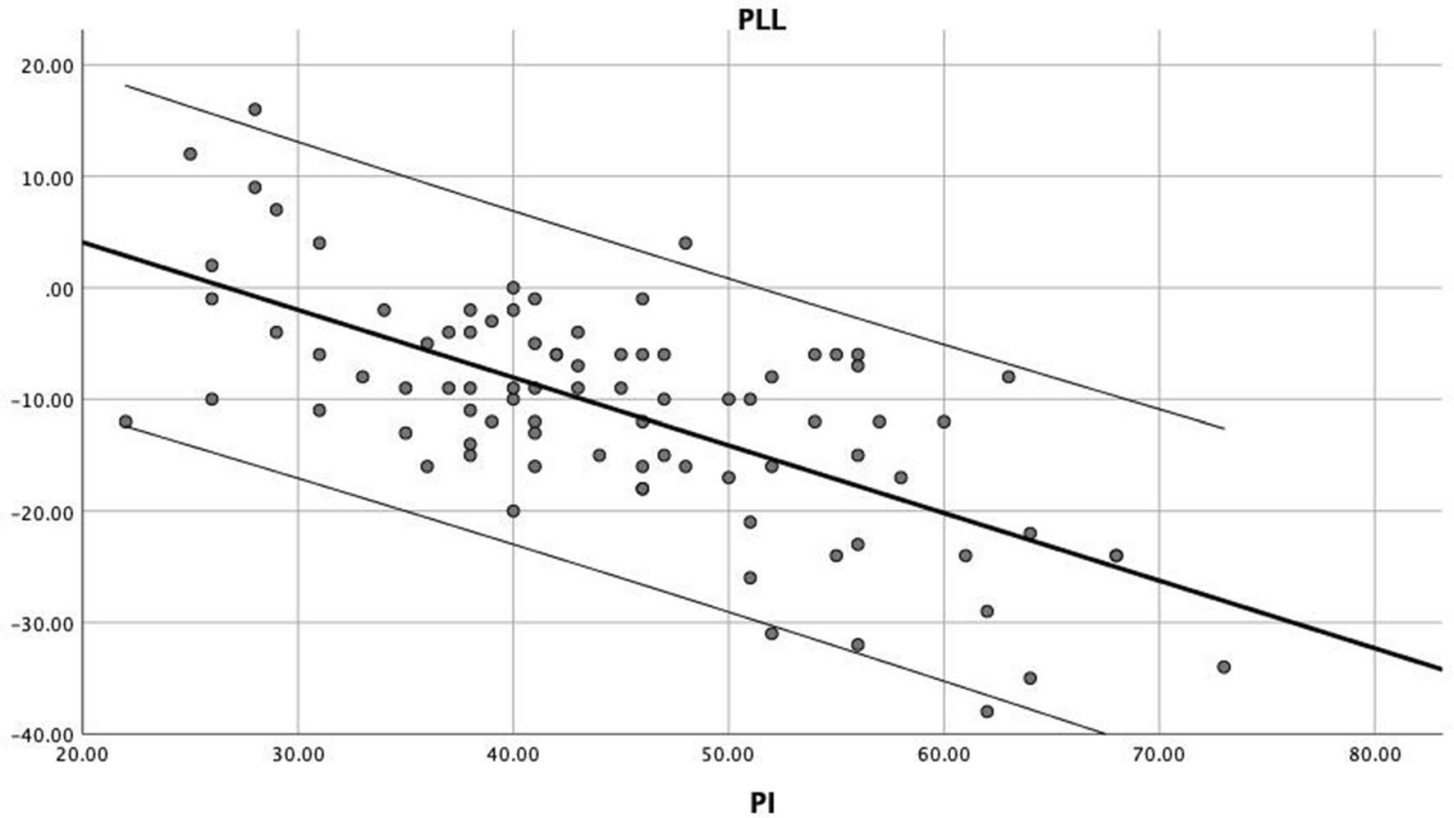
Linear stepwise regression between proximal lumbar lordosis and pelvic incidence (R^2^ = 0.452, PLL = 16.2–0.61 * PI, *p* < 0.001). PLL, proximal lumbar lordosis; PI, pelvic incidence.

PLL revealed a correlation with lumbar lordosis apex (r = 0.603, *p* = 0.002) and inflexion point (r = 0.536, *p* = 0.004), but did not DLL with LL apex (r = 0.204, *p* = 0.058) or inflexion point (r = 0.210, *p* = 0.051) (Table 2). PI revealed a greater correlation with PLL (r = −0.673, *p* < 0.001) than with DLL (r = −0.237, *p* = 0.045). Linear stepwise regression analysis also exhibited the correlation between PI and PLL (R^2^ = 0.452, PLL = 16.2–0.61*PI, *p* < 0.001) (Fig. 4). PI also correlated with LL apex (r = −0.675, *p* < 0.001), inflexion point (r = −0.573, *p* < 0.001), and ES muscularity (r = 0.307, *p* = 0.007), but without MF muscularity (r = 0.013, *p* = 0.940). ES muscularity correlated with LL apex (r = −0.279, *p* = 0.014) and inflexion point (r = −0.227, *p* = 0.047) (Table 2).

**Table 2:** Pearson correlation analysis of lumbar morphology, pelvic incidence and paraspinal muscles.

Parameters	PLL	DLL	LL Apex	Inflexion point	ES muscularity	MF muscularity
PI	− 0.673^‡^	− 0.237^†^	− 0.675^‡^	− 0.573^‡^	0.307^‡^	0.013
PLL	–	0.300^†^	0.603^‡^	0.536^‡^	− 0.179	− 0.031
DLL		–	0.204	0.210	− 0.092	− 0.067
LL Apex			–	0.704^‡^	− 0.279^‡^	0.017
Inflexion point				–	− 0.227^†^	0.072
ES muscularity					–	0.580^‡^

*PI* pelvic incidence, *PLL* proximal lumbar lordosis, *DLL* distal lumbar lordosis, *LL Apex* lumbar lordosis apex, *ES* erector spinae, *MF* multifidus.

^†^*p* < 0.05.

^‡^*p* < 0.01.

## Discussion

A deep understanding of lumbar lordosis morphology is helpful in clinical practice for restoring normal sagittal spinal alignment in long fusion surgeries^12,18–21^. Roussouly and colleagues designed a detailed classification of normal sagittal spine profile in Caucasian population according to PI and SS value^10,11^. Ethnicity plays an important role in sagittal alignment^22^. Since the entire cohort in the present study was from Asian population, the distribution of Roussouly classification was different from previous studies^10^. There were more cases in type 1 and type 2, but less in type 3 and type 4 in the present study than the previous study^10^.

There was also a different distribution of PI value. Much more cases possessed a PI < 45°, but less with PI > 60° than Caucasian population^11,12^. It was comparable to Asian population according to previous reports^3,4^. Thus, the cohort was stratified into 4 groups rather than 3 groups, according to PI value: very low PI group (PI < 30°), low PI group (PI = 30°–45°), moderate PI group (45°–60°), and high PI group (PI > 60°).

**Figure 5:**
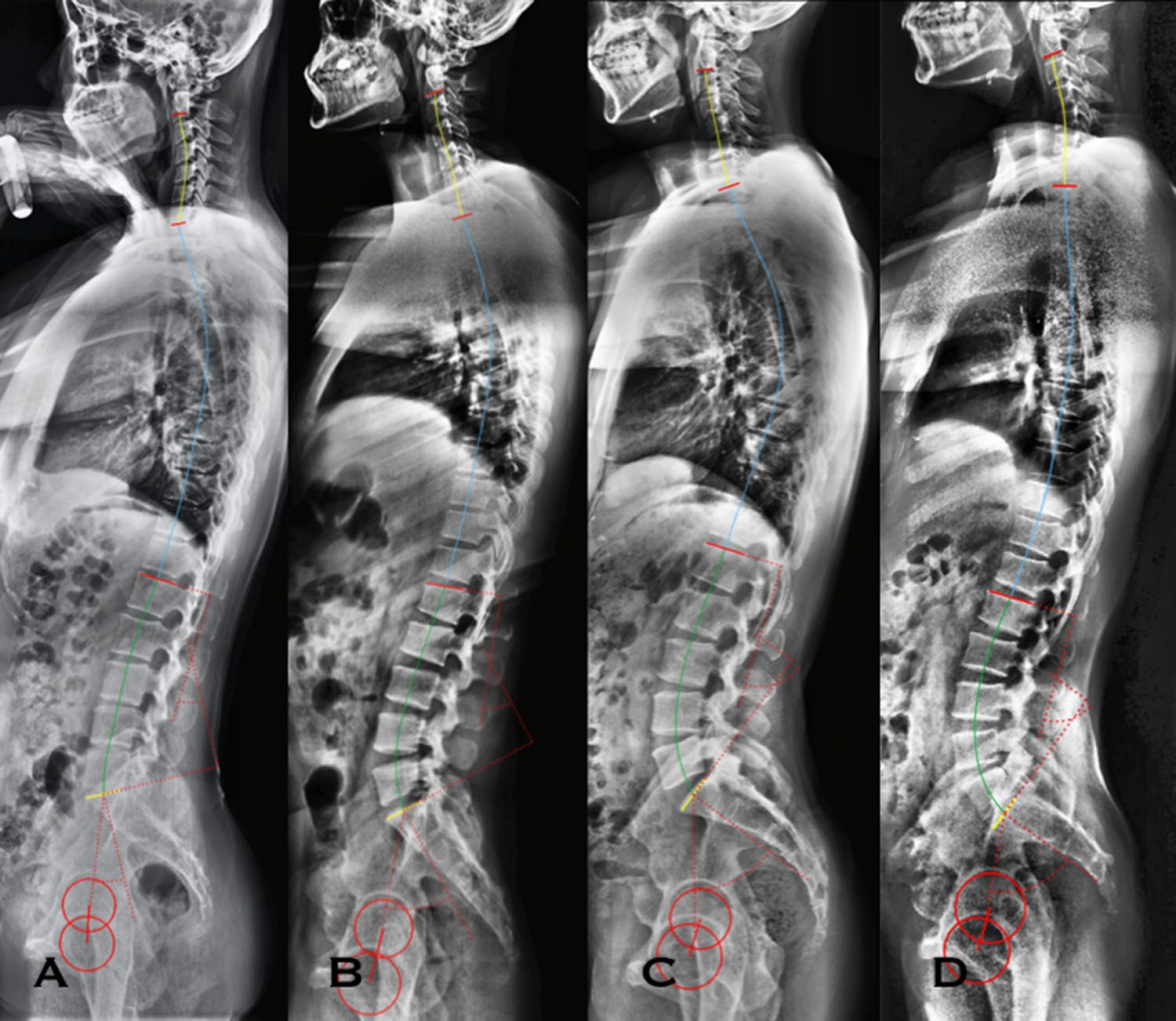
The graphs show the correlation between pelvic incidence and lumbar lordosis morphology. Across the stratification by PI value, from very low PI to high PI group, lumbar lordosis increases, and lumbar lordosis apex migrates higher. A with very low PI, PI = 21°, LL apex = L5/S1, LL = −30°, PLL = −1°; B with low PI, PI = 39°, LL apex = L4/5, LL = −38°, PLL = −7°. C with moderate PI, PI = 53.0°, LL apex = L4, LL = −60°, PLL = −20°. D with high PI, PI = 64°, LL apex = L3/4, LL = −70°, PLL = −28°. *PI* pelvic incidence, *LL* apex, lumbar lordosis apex, *LL* lumbar lordosis, *PLL* proximal lumbar lordosis.

Across the stratification by PI value, not only LL magnitude increases, but also location of LL apex and inflexion point migrates higher (Fig. 5). The results correspond with the previous studies^5,10,11^. According to the Pearson correlation analysis in the present study, PLL is more variable accommodating to PI than DLL. PLL increases across the groups, but DLL maintains constant between the low PI group and moderate PI group, the majority of the entire cohort (78%, 68/87). It illustrates that PLL plays a more important role than DLL in adjusting LL morphology to PI.

It is also demonstrated by the results of Pearson correlation analysis in the present study. PLL exhibits a correlation with LL apex and inflexion joint location, but DLL does not have such a statistically significant correlation. It supports the conclusion in Pesenti and colleagues’ study that PLL is the driver of compensatory mechanism of LL morphology to PI^12^.

ES muscularity also plays a role in adjusting LL morphology to PI, which is exhibited by the results of Pearson correlation analysis. ES might be one compensatory mechanism of LL morphology accommodating to PI. Several researches in elder patients demonstrate that lumbar muscularity correlates with LL morphology^14,15^. ES muscularity also correlates with the proximal junctional kyphosis (PJK) risk after long fusion surgery^23^. All the results support the assumption above.

The results are helpful for restoring a rational lumbar lordosis shape in long fusion surgery. For a patient with low PI value, a rational LL shape should be restored with a lower LL apex, a smaller PLL, but a same sized DLL, compared to a patient with moderate PI^18^. Otherwise, it is not suitable to restore a greater PLL, a higher LL apex for patient with low PI, because the lower ES muscularity could not provide enough strength to balance an excessive LL^23^. As a result, proximal junctional kyphosis complication might occur after long fusion surgery.

## Data Availability

Data and materials reviewed in the study are available.

## References

[1] Mac-Thiong JM, Roussouly P, Berthonnaud E, Guigui P (2010). Sagittal parameters of global spinal balance: normative values from a prospective cohort of seven hundred nine Caucasian asymptomatic adults. Spine (Phila Pa 1976)..

[2] Mac-Thiong JM, Roussouly P, Berthonnaud E, Guigui P (2011). Age- and sex-related variations in sagittal sacropelvic morphology and balance in asymptomatic adults. Eur. Spine J..

[3] Yukawa Y (2018). Normative data for parameters of sagittal spinal alignment in healthy subjects: an analysis of gender specific differences and changes with aging in 626 asymptomatic individuals. Eur. Spine J..

[4] Yokoyama K (2017). Age-related variations in global spinal alignment and sagittal balance in asymptomatic Japanese adults. Neurol. Res..

[5] Pan C, Wang G, Sun J (2020). Correlation between the apex of lumbar lordosis and pelvic incidence in asymptomatic adult. Eur. Spine J..

[6] Mangone M (2020). Sagittal spinal alignment in patients with ankylosing spondylitis by rasterstereographic back shape analysis: an observational retrospective study. Eur. J. Phys. Rehab. Med..

[7] Mangone M (2019). Changes in spine alignment and postural balance after breast cancer surgery: a rehabilitative point of view. BioRes. Open Access..

[8] Paolucci T (2020). Straighten your back! self-correction posture and postural balance in “non-rehabilitative instructed” multiple sclerosis patients. Neurorehabilitation..

[9] Roussouly P, Berthonnaud E, Dimnet J (2003). Geometrical and mechanical analysis of lumbar lordosis in an asymptomatic population: proposed classification. Revue De Chirurgie Orthopedique et Reparatrice De Lapparl Moteur..

[10] Roussouly P, Gollogly S, Berthonnaud E, Dimnet J (2005). Classification of the normal variation in the sagittal alignment of the human lumbar spine and pelvis in the standing position. Spine (Phila Pa 1976)..

[11] Laouissat F, Sebaaly A, Gehrchen M, Roussouly P (2018). Classification of normal sagittal spine alignment: refounding the Roussouly classification. Eur. Spine J..

[12] Pesenti S (2018). The amount of proximal lumbar lordosis is related to pelvic incidence. Clin. Orthop. Relat. Res..

[13] Toppenberg RM, Bullock MI (1986). The interrelation of spinal curves, pelvic tilt and muscle lengths in the adolescent female. Aust J Physiother..

[14] Belavy DL, Armbrecht G, Richardson CA, Felsenberg D, Hides JA (2011). Muscle atrophy and changes in spinal morphology: is the lumbar spine vulnerable after prolonged bed-rest?. Spine (Phila Pa 1976)..

[15] Jun HS (2016). The effect of lumbar spinal muscle on spinal sagittal alignment: evaluating muscle quantity and quality. Neurosurgery..

[16] Yagi M, Fujita N, Okada E, Tsuji O, Watanabe K (2018). Surgical outcomes for drop body syndrome in adult spinal deformity. Spine..

[17] Kang CH, Shin MJ, Kim SM, Lee SH, Lee CS (2007). MRI of paraspinal muscles in lumbar degenerative kyphosis patients and control patients with chronic low back pain. Clin. Radiol..

[18] Pizones J (2020). Restoring the ideal Roussouly sagittal profile in adult scoliosis surgery decreases the risk of mechanical complications. Eur. Spine J..

[19] Damiani C (2020). Trade-offs with rehabilitation effectiveness (Res) and efficiency (Rey) in a sample of italian disabled persons in a in post-acuity rehabilitation unit. Ann Ig..

[20] Seccia R (2020). Data of patients undergoing rehabilitation programs. Data Brief..

[21] Yan P, Bao H, Qiu Y, Bao M, Zhu F (2016). Mismatch between proximal rod contouring and proximal junctional angle: a predisposed risk factor for proximal junctional kyphosis in degenerative scoliosis. Spine..

[22] Diebo BG (2016). Role of ethnicity in alignment compensation: propensity matched analysis of differential compensatory mechanism recruitment patterns for sagittal malalignment in 288 ASD patients from Japan Korea and United States. Spine.

[23] Hyun SJ, Kim YJ, Rhim SC (2016). Patients with proximal junctional kyphosis after stopping at thoracolumbar junction have lower muscularity, fatty degeneration at the thoracolumbar area. Spine J..

